# Supplementary material for: Modeling glioblastoma heterogeneity as a dynamic network of cell states
Source: Mol Syst Biol. 2021 Sep 16;17(9):e10105. doi: 10.15252/msb.202010105 (PMC8444284; doi:10.15252/msb.202010105)
Supplement: Supplementary file 6 — Source Data for Figure 5 [file MSB-17-e10105-s004.zip › Figure5A_sourcedata/GSEA_3017/nosigdb_stateE.GseaPreranked.1622795031093/RICHARDS_NOWAKOWSKI_SCIENCE_2017_VRG_UPREG.html]

Details for gene set RICHARDS\_NOWAKOWSKI\_SCIENCE\_2017\_VRG\_UPREG[GSEA]

|  || Dataset | state63017 |
| Phenotype | NoPhenotypeAvailable |
| Upregulated in class | na\_pos |
| GeneSet | RICHARDS\_NOWAKOWSKI\_SCIENCE\_2017\_VRG\_UPREG |
| Enrichment Score (ES) | 0.5141776 |
| Normalized Enrichment Score (NES) | 3.7001054 |
| Nominal p-value | 0.0 |
| FDR q-value | 0.0 |
| FWER p-Value | 0.0 |
Table: GSEA Results Summary

  

Fig 1: Enrichment plot: RICHARDS\_NOWAKOWSKI\_SCIENCE\_2017\_VRG\_UPREG      
 Profile of the Running ES Score & Positions of GeneSet Members on the Rank Ordered List

  

| PROBE | GENE SYMBOL | GENE\_TITLE | RANK IN GENE LIST | RANK METRIC SCORE | RUNNING ES | CORE ENRICHMENT || 1 | GFAP |  |  | 1 | 1.383 | 0.0395 | Yes |
| 2 | CYR61 |  |  | 3 | 1.169 | 0.0727 | Yes |
| 3 | FABP7 |  |  | 11 | 0.920 | 0.0917 | Yes |
| 4 | ANXA2 |  |  | 18 | 0.733 | 0.1063 | Yes |
| 5 | TTYH1 |  |  | 19 | 0.731 | 0.1278 | Yes |
| 6 | CNN3 |  |  | 24 | 0.702 | 0.1438 | Yes |
| 7 | LGALS3 |  |  | 25 | 0.697 | 0.1643 | Yes |
| 8 | IFITM3 |  |  | 26 | 0.682 | 0.1843 | Yes |
| 9 | MSMO1 |  |  | 28 | 0.648 | 0.2022 | Yes |
| 10 | TAGLN2 |  |  | 32 | 0.621 | 0.2170 | Yes |
| 11 | RAB31 |  |  | 35 | 0.598 | 0.2323 | Yes |
| 12 | HMGCS1 |  |  | 37 | 0.593 | 0.2486 | Yes |
| 13 | CALD1 |  |  | 41 | 0.580 | 0.2622 | Yes |
| 14 | C1orf61 |  |  | 43 | 0.553 | 0.2772 | Yes |
| 15 | F3 |  |  | 55 | 0.506 | 0.2795 | Yes |
| 16 | MRC2 |  |  | 56 | 0.505 | 0.2943 | Yes |
| 17 | SLC1A3 |  |  | 66 | 0.472 | 0.2978 | Yes |
| 18 | SRGAP3 |  |  | 67 | 0.467 | 0.3115 | Yes |
| 19 | PDPN |  |  | 79 | 0.438 | 0.3117 | Yes |
| 20 | PPP1CB |  |  | 80 | 0.438 | 0.3246 | Yes |
| 21 | DOK5 |  |  | 81 | 0.437 | 0.3374 | Yes |
| 22 | SIPA1L1 |  |  | 84 | 0.429 | 0.3477 | Yes |
| 23 | CST3 |  |  | 86 | 0.425 | 0.3590 | Yes |
| 24 | NES |  |  | 92 | 0.414 | 0.3654 | Yes |
| 25 | EFNB2 |  |  | 94 | 0.411 | 0.3764 | Yes |
| 26 | SHROOM3 |  |  | 95 | 0.410 | 0.3884 | Yes |
| 27 | EZR |  |  | 96 | 0.410 | 0.4005 | Yes |
| 28 | STK17A |  |  | 97 | 0.408 | 0.4125 | Yes |
| 29 | GPM6B |  |  | 105 | 0.391 | 0.4159 | Yes |
| 30 | CD99 |  |  | 107 | 0.389 | 0.4262 | Yes |
| 31 | CLU |  |  | 119 | 0.364 | 0.4242 | Yes |
| 32 | SEMA6A |  |  | 130 | 0.348 | 0.4229 | Yes |
| 33 | ARL6IP5 |  |  | 134 | 0.345 | 0.4296 | Yes |
| 34 | GATM |  |  | 149 | 0.335 | 0.4233 | Yes |
| 35 | ZFP36L2 |  |  | 162 | 0.324 | 0.4190 | Yes |
| 36 | SQLE |  |  | 168 | 0.319 | 0.4226 | Yes |
| 37 | DDAH1 |  |  | 170 | 0.317 | 0.4308 | Yes |
| 38 | JUN |  |  | 172 | 0.316 | 0.4389 | Yes |
| 39 | LRRC17 |  |  | 182 | 0.310 | 0.4377 | Yes |
| 40 | SLC35F1 |  |  | 183 | 0.309 | 0.4467 | Yes |
| 41 | BICD1 |  |  | 198 | 0.297 | 0.4393 | Yes |
| 42 | DKK3 |  |  | 205 | 0.293 | 0.4410 | Yes |
| 43 | SAT1 |  |  | 206 | 0.291 | 0.4496 | Yes |
| 44 | GNG12 |  |  | 212 | 0.287 | 0.4523 | Yes |
| 45 | FERMT2 |  |  | 219 | 0.283 | 0.4537 | Yes |
| 46 | TMEM47 |  |  | 222 | 0.282 | 0.4597 | Yes |
| 47 | SAMD4A |  |  | 224 | 0.281 | 0.4667 | Yes |
| 48 | NPAS3 |  |  | 227 | 0.280 | 0.4727 | Yes |
| 49 | DAG1 |  |  | 228 | 0.279 | 0.4809 | Yes |
| 50 | ITGB8 |  |  | 239 | 0.268 | 0.4772 | Yes |
| 51 | QKI |  |  | 241 | 0.267 | 0.4839 | Yes |
| 52 | CHMP2A |  |  | 243 | 0.266 | 0.4906 | Yes |
| 53 | GPC4 |  |  | 246 | 0.264 | 0.4960 | Yes |
| 54 | HMGCR |  |  | 251 | 0.261 | 0.4991 | Yes |
| 55 | INSIG1 |  |  | 252 | 0.261 | 0.5068 | Yes |
| 56 | ATP1B2 |  |  | 260 | 0.258 | 0.5063 | Yes |
| 57 | FDFT1 |  |  | 266 | 0.252 | 0.5079 | Yes |
| 58 | MOXD1 |  |  | 268 | 0.252 | 0.5142 | Yes |
| 59 | ZFP36L1 |  |  | 287 | -0.255 | 0.5009 | No |
| 60 | ACBD5 |  |  | 307 | -0.259 | 0.4867 | No |
| 61 | TGIF1 |  |  | 318 | -0.263 | 0.4829 | No |
| 62 | CA12 |  |  | 325 | -0.263 | 0.4837 | No |
| 63 | CNTLN |  |  | 326 | -0.264 | 0.4914 | No |
| 64 | SLC12A2 |  |  | 389 | -0.279 | 0.4282 | No |
| 65 | CTBP2 |  |  | 393 | -0.280 | 0.4330 | No |
| 66 | MAGI1 |  |  | 413 | -0.287 | 0.4195 | No |
| 67 | PPA1 |  |  | 420 | -0.289 | 0.4211 | No |
| 68 | NUMA1 |  |  | 477 | -0.307 | 0.3656 | No |
| 69 | BARD1 |  |  | 510 | -0.318 | 0.3381 | No |
| 70 | JADE1 |  |  | 543 | -0.329 | 0.3109 | No |
| 71 | ETV1 |  |  | 563 | -0.341 | 0.2990 | No |
| 72 | CACYBP |  |  | 589 | -0.352 | 0.2806 | No |
| 73 | PHIP |  |  | 643 | -0.384 | 0.2308 | No |
| 74 | ZEB1 |  |  | 653 | -0.396 | 0.2321 | No |
| 75 | EGR1 |  |  | 656 | -0.398 | 0.2415 | No |
| 76 | PSAT1 |  |  | 720 | -0.448 | 0.1821 | No |
| 77 | DBI |  |  | 776 | -0.526 | 0.1341 | No |
| 78 | SYNE2 |  |  | 859 | -0.728 | 0.0611 | No |
| 79 | FABP5 |  |  | 939 | -1.294 | 0.0081 | No |
Table: GSEA details [plain text format]

  

Fig 2: RICHARDS\_NOWAKOWSKI\_SCIENCE\_2017\_VRG\_UPREG: Random ES distribution      
 Gene set null distribution of ES for **RICHARDS\_NOWAKOWSKI\_SCIENCE\_2017\_VRG\_UPREG**
